# Anti-Inflammatory Properties and Chemical Characterization of the Essential Oils of Four *Citrus* Species

**DOI:** 10.1371/journal.pone.0153643

**Published:** 2016-04-18

**Authors:** Jorge Luis Amorim, Daniel Luiz Reis Simas, Mariana Martins Gomes Pinheiro, Daniela Sales Alviano Moreno, Celuta Sales Alviano, Antonio Jorge Ribeiro da Silva, Patricia Dias Fernandes

**Affiliations:** 1 Universidade Federal do Rio de Janeiro, Instituto de Ciências Biomédicas, Laboratório de Farmacologia da Dor e da Inflamação, Rio de Janeiro, Brasil; 2 Universidade Federal do Rio de Janeiro, Instituto de Pesquisa de Produtos Naturais, Laboratório de Análise Fitoquímica, Rio de Janeiro, Brasil; 3 Universidade Federal do Rio de Janeiro, Instituto de Microbiologia Paulo de Góes, Laboratório de Estruturas de Superfície de Micro-organismos, Rio de Janeiro, Brasil; National Institutes of Health, UNITED STATES

## Abstract

Citrus fruits have potential health-promoting properties and their essential oils have long been used in several applications. Due to biological effects described to some citrus species in this study our objectives were to analyze and compare the phytochemical composition and evaluate the anti-inflammatory effect of essential oils (EO) obtained from four different *Citrus* species. Mice were treated with EO obtained from *C*. *limon*, *C*. *latifolia*, *C*. *aurantifolia* or *C*. *limonia* (10 to 100 mg/kg, p.o.) and their anti-inflammatory effects were evaluated in chemical induced inflammation (formalin-induced licking response) and carrageenan-induced inflammation in the subcutaneous air pouch model. A possible antinociceptive effect was evaluated in the hot plate model. Phytochemical analyses indicated the presence of geranial, limonene, γ-terpinene and others. EOs from *C*. *limon*, *C*. *aurantifolia* and *C*. *limonia* exhibited anti-inflammatory effects by reducing cell migration, cytokine production and protein extravasation induced by carrageenan. These effects were also obtained with similar amounts of pure limonene. It was also observed that *C*. *aurantifolia* induced myelotoxicity in mice. Anti-inflammatory effect of *C*. *limon* and *C*. *limonia* is probably due to their large quantities of limonene, while the myelotoxicity observed with *C*. *aurantifolia* is most likely due to the high concentration of citral. Our results indicate that these EOs from *C*. *limon*, *C*. *aurantifolia* and *C*. *limonia* have a significant anti-inflammatory effect; however, care should be taken with *C*. *aurantifolia*.

## Introduction

As rich sources of dietary fiber, vitamin C, phenols, and flavonoids, *Citrus* fruits are believed to have potential health-promoting properties [1]. Essential oils of *Citrus* species have long been used for insecticidal, medicinal and cosmetic applications [2]. The peel (pericarp) essential oils of various species of *Citrus* are widely used as flavoring agents in food, beverages and confections [3]. Recent studies point to the possibility of employing *Citrus* oils or their active principles to prevent or treat several pathological conditions, where they can be used as antimicrobial [4], antifungal [5], neuroprotective [6], anxiolytic, anticonvulsant and sedative [7], antinociceptive [8], anti-inflammatory [9] and antioxidant [10] agents.

The chemical composition of the peel essential oils of *Citrus* fruits is complex and rich in limonene and other monoterpene components, such as β-pinene and γ-terpinene, as well as some sesquiterpenes generally present in low amounts [11]. According to Mehl et al. [12], the volatile fraction (85% to 99% of liquid oil) is composed predominantly of terpenes (monoterpenes and sesquiterpenes) and their oxygenated derivatives, as well as aliphatic alcohols, esters and aldehydes. Limonene is the main component of the essential oils of *Citrus* (30% to 70% in different varieties). Other significant components include α and β-pinene, γ-terpinene, terpinolene, sabinene.

Due to the great variety of species distributed all over the planet and the large number of widely used species in Brazil this study seeks to assess the chemical composition and the anti-inflammatory properties of the essential peel oils of the fruits *C*. *limon*, *C*. *aurantifolia*, *C*. *limonia* and *C*. *latifolia*. We also investigate possible existing toxic effects after the use of these species.

## Methods

### Plant material

*C*. *limon* [(L.) Osbeck, “siciliano” lemon], *C*. *latifolia* (Tanaka ex Q. Jimenez, “tahiti” lime), *C*. *aurantifolia* [(Christm.) Swingle, “mirim” lime] and *C*. *limonia* [(L.) Osbeck, “cravo” lime) were collected in the farm of Instituto Vital Brazil, Rio de Janeiro, Brazil, located in Cachoeira de Macacu, Road Rio-Friburgo Km 32.5. Via Magé, RJ 122. GPS coordenatiors are 22° 20’ 753” S, 42° 42’ 858” W. The botanical identification was provided by Dr. Rosana Conrado Lopes (Biology Institute, Federal University of Rio de Janeiro/Brazil), and voucher specimens are deposited in the RFA Herbarium, Federal University of Rio de Janeiro/Brazil. The exsiccate numbers are: *C*. *limonia* (RFA-39493), *C*. *aurantifolia* (RFA-39492), *C*. *limon* (RFA-39494) and *C*. *latifolia* (RFA-39491).

The fruit peels were manually removed and homogenized with water in a blender, then immediately submitted to hydro distillation in a modified Clevenger-type apparatus for 2 hours. After extraction, the oils were dried over anhydrous Na_2_SO_4_ and stored at -18°C. Typically, 150 g of fruit peels were used and average yields of 1.4% were obtained.

### GC/FID analysis

GC analyses were carried out using a Shimadzu GC 2010 (Tokyo, Japan) equipped with a flame ionization detector on a DB5 fused silica capillary column (30 m, 0.25 mm i.d., film thickness 0.25 μm). The oven temperature was programmed to rise from 60°C to 246°C at 3°C/min, then hold at 246°C for 20 min. Injector and detector temperatures were maintained at 220°C and 290°C, respectively. The oil samples were dissolved in CHCl_3_, and 1 μL aliquots were injected in split mode with split ratio of 1:33 using H_2_ as the carrier gas (1.44 mL/min). Kovats retention indices (KI) of the compounds were determined relative to the retention times of a series of n-alkanes (C7–C30) with linear interpolation. The relative amounts of the components were calculated based on GC peak areas without correction factors.

### GC-MS analysis

Analyses were made on a Shimadzu QP2010 Plus system using a DB5-MS fused silica capillary column (30 m, 0.25 mm I.D., 0.25 μm film thickness). The oven temperature was programmed to rise from 60°C to 246°C at 3°C/min and then to hold at this temperature for 20 min. The carrier gas was He (99.999%) with a flow rate of 1.03 mL/min and the injector temperature was 220°C. Oil samples were diluted in CHCl_3_ and 1 μL aliquots were injected in split mode (split ratio 1:50). Mass spectra were obtained under electroionization at 70 eV with a mass range m/z of 40 to 1000 D.

### Compound identification

The identification of individual components was based on (i) a comparison of the mass spectral fragmentation patterns with those stored in the NIST Mass Spectral Library and (ii) a comparison of the GC Kovats retention indices (KI) on a DB-5 column with those of authentic compounds and from literature data [13]. Geranial and neral were additionally identified by co-injection of authentic standards obtained by preparative liquid chromatography RP18 column (Supelco, 150 x 10 mm; isocratic condition, mobile phase: methanol:water 60/40 and UV detector at 254 nm) from citral. The characterization of the isolated compounds was made by comparison of their ^13^C NMR spectra (CDCl_3_, 100 MHz) with literature data [14].

### Animals

Male Swiss Webster mice (2 months old, 18–25 g), kindly donated by Instituto Vital Brazil, were used in this study. Animals were housed in a temperature-controlled room at 22 ± 2°C with a 12 h light/dark cycle and free access to pelleted food (Nutrilab, Brazil) and water. Twelve hours before each experiment, the animals received only water in order to avoid food interference with substances absorption. The animals were acclimatized to the laboratory for at least 1 h before testing and were used only once throughout the experiments. Physical condition of animals was daily monitored and animals with any signs of suffering were euthanized. Also none of animals used became severely ill or died at any time prior to the experimental endpoint.

The research was conducted in accordance with the internationally accepted principles for laboratory animal use and care. The experimental protocols used in this work followed the rules advocated by Law 11,794, of October 8, 2008 by the National Council of Animal Experimentation Control (CONCEA) and were approved by the Ethics Committee of Animal Use (CEUA), Science Center Health/UFRJ and received the number DFBCICB015-04/16.

### Drugs, reagents and treatments

All solvents were with chromatographic grade (Tedia, Brazil). Acetylsalicylic acid (ASA), carrageenan, dexamethasone, citral and limonene were purchased from Sigma-Aldrich (St. Louis, MO, U.S.A.). Formalin was purchased from Merck (Germany). Morphine sulfate was kindly provided by Cristália (São Paulo, Brazil). Essential oils (EO) were dissolved in oil (Sigma-Aldrich, USA) to prepare a stock solution at 100 mg/ml. They were administered by oral gavage, at doses of 10 to 100 mg/kg 60 min prior to experiments. Morphine (5 mg/kg, p.o.), ASA (100 mg/kg, p.o.) and dexamethasone (1.5 mg/kg, i.p.) were used as reference drugs. All drugs were diluted in phosphate buffer saline (PBS) just before use. The control group was composed by vehicle (PBS with the same amount of oil used in the highest dose). The final concentration of oil did not exceed 0.5%, and had no effect *per se*. The doses of ASA and morphine were chosen based on previous experiments performed by our group and caused a 50% reduction on each protocol (IC_50_).

### Hot plate test

Mice were tested according to the method described by Sahley and Berntson [15]^5^ and adapted by Matheus et al. [16]. Animals were placed on a hot plate (Insight Equipment, Brazil) set at 55 ± 1°C. At successive intervals of 30 min after oral administration of EOs or vehicle, the reaction time was recorded when the animals licked their fore- and hind-paws and jumped. Baseline was considered the mean reaction time obtained at 60 and 30 min before administration of the compounds, vehicle, or morphine and was defined as the normal reaction of the animal to the temperature. When animals were kept on the hot plate for a period of time greater than three times the baseline (cut-off), they were removed to avoid possible damage to the paws. Antinociception was quantified as either the increase in baseline (%) calculated by the formula (reaction time x 100/baseline)– 100.

### Formalin test

The licking behavior was examined immediately after injection of formalin into the hind paw. The procedure was similar to the method described by Hunskaar and Hole [17] and adapted by Gomes et al. [18]. Mice received an injection of 20 μL of formalin (2.5% v/v) into the dorsal surface of the left hind paw. The time that the animal spent licking the injected paw was immediately recorded. The nociceptive and inflammatory response consists of the following two phases: the first phase lasts until 5 min after the formalin injection (first phase, neurogenic pain response), and the second phase occurs 15–30 min after the formalin injection (second phase, inflammatory pain response). The animals were pre-treated with oral doses of EOs, vehicle or ASA for 60 min before the administration of formalin.

### Subcutaneous air pouch (SAP) model

Animals were used as described by Sedgwick et al. [19] with modifications done by Raymundo and colleagues [20]. After subcutaneous injection of 10 mL of sterile air in the dorsal region in three alternate days, an injection of sterile carrageenan suspension (1%; 1 mL) was done in the SAP formed. Mice were divided in the following groups: vehicle, EOs (10, 30 or 100 mg/kg, p.o.), dexamethasone (1.5 mg/kg, i.p.) treated groups 1 h before carrageenan injection. Another vehicle-treated group was used in mice that received PBS (phosphate buffer saline, 1 mL) in SAP. After 24 h all groups were sacrificed, SAP was washed with 1 mL of sterile PBS and exudates collected. Bone marrow cells were obtained by flushing the femoral cavity with 1 mL of phosphate buffer saline (PBS). Peripheral blood was collected in a heparinized tube. Cells counts in from aliquot of bone marrow cells, blood cells suspension or exudates were determined in a CellPocH-100iV Diff (Sysmex) haematology analyser. The exudates were also centrifuged (5,000 x *g*, 10 min, 4°C) and aliquots of the supernatants were stored at -20°C until the assays.

### Quantification of TNF-α, IL-1β, IFN-γ and protein

Supernatants from the exudates collected from the SAP were used to measure tumor necrosis factor-α (TNF-α), interleukin 1β (IL-1β), interferon-γ (IFN-γ) by enzyme-linked immunosorbent assay (ELISA) according to the manufacturer’s instructions (B&D, USA) and extravasated protein determined using the BCA method (BCA™ Protein Assay Kit, Pierce).

### Nitrate measurement

To evaluate the production of nitric oxide (NO), the nitrate accumulated in the SAP exudates was measured according to the method described by Bartholomew [21] and adapted by Raymundo et al [20]. The nitrite concentrations obtained after nitrate conversion were measured by mixing equal parts of sample and Griess Reagent (1% sulphanilamide, 0.1% *N-*(1-Naphthyl) ethylenediamine dihydrochloride, 10% H_3_PO_4_) [22]. FlexStation microplate reader (Molecular Devices, USA) was used to measure the absorbance at 540 nm. Nitrite concentration was measured by comparison with a standard curve of sodium nitrite.

### Behavioral and stomach observations

To evaluate a possible toxic effect we adapted the method used by Lorke [23]. Briefly, different groups of mice were treated with 500 mg/kg of each EO. During 5 consecutive days several parameters (*i*.*e*., convulsion, sedation, respiration, and food and water intake) were observed. After five days the animals were euthanized with an overdose of ketamine/xilazine (150 mg/10 mg/kg). The stomachs of the animals were removed and opened to observe any signs of hyperemia and the presence or absence of ulcer.

### Statistical analysis

All experimental groups consisted of 6–10 mice. The results are presented as the mean ± S.D. Statistical significance between groups was performed by applying analysis of variance (ANOVA) followed by Bonferroni’s test. *P* values less than 0.05 (*p* < 0.05) were considered significant.

## Results

### Chemical characterization of essential oils

Table 1 show retention indices and percentages of each compound identified in the essential oils of the *Citrus* species studied. Twenty-one to thirty-one compounds were identified, comprising 94.8 to 99.4% of the oils. Limonene (31.1 to 65.7%), β-pinene (5.1 to 13.1%) and γ-terpinene (10.8 to 12.2%) were the compounds found in highest concentration. The oils of these species are notable for possessing relatively low levels of limonene compared to oranges (less than 70%) and relatively high levels of *α*-pinene, *β*-pinene, sabinene and *γ*-terpinene [24]. The sesquiterpenes β-bisabolene, trans-α-bergamotene and β-caryophyllene were present in the essential oils of all the citrus studied in low amounts. The majority of compounds identified are hydrocarbon monoterpenes. Kovats indexes (KI) were compared with literature [25].

**Table 1:** Chemical composition of peel essential oils of four *Citrus* samples.

Compound	KI cal.	KI lit.	*C*. *limon*	*C*. *aurantifolia*	*C*. *limonia*	*C*. *latifolia*
			Percent relative area			
α-thujene	930	932	0.7	0.1	0.4	0.2
α-pinene	939	939	0.7	0.6	2	1.5
camphene	954	954	0.1	-	-	0.3
sabinene	975	978	3.4	1.3	1.2	-
β-pinene	979	982	13.1	8.5	9	5.1
myrcene	991	992	2.7	1	1.9	1.6
α-phellandrene	1003	1004	-	-	-	0.2
α-terpinene	1017	1019	0.5	0.3	0.4	0.6
r-cymene	1026	1028	0.3	0.5	0.7	0.1
limonene	1029	1033	53.9	31.1	65.7	35.4
β-cymene	1050	1051	-	-	0.1	0.2
γ-terpinene	1060	1062	12.2	10.8	12.3	12.1
terpinolene	1089	1090	0.8	0.8	0.7	1.4
linalool	1097	1099	0.3	1.1	0.2	1.7
nonanal	1101	-	0.2	-	-	-
citronelal	1153	1153	-	-	0.3	-
terpinen-4-ol	1177	1178	0.5	1.6	1	2.3
α-terpineol	1189	1190	0.8	1.6	1.7	6.5
n-decanal	1202	1204	-	0.9	0.1	-
nerol	1230	1229	0.2	1.1	-	1.1
neral	1238	1238	1.7	7.1	-	2.6
geraniol	1253	1256	0.3	1.8	-	1.3
geranial	1267	1271	2.2	9.6	-	3.6
δ-elemene	1338	1339		0.5	-	0.2
neryl acetate	1362	1365	0.8	0.2	-	2.3
geranil acetate	1381	1381	0.4	-	-	0.8
β-elemene	1391	1392	-	0.3	-	-
cis-α-bergamotene	1413	1415	-	-	-	0.2
β-caryophyllene	1419	1420	0.3	2.4	0.2	2
trans-α-bergamotene	1435	1437	0.4	2.1	0.4	3.4
α-humulene	1455	1454	-	0.3	-	0.2
(E)-β-farnesene	1458	1458	-	0.2	-	-
germacrene D	1485	1481	-	0.8	0.2	-
α-bisabolene	1506	1506	-	-	-	0.6
β-bisabolene	1506	1509	0.6	6.8	0.7	6.5
δ-cadinene	1523	1523	-	0.6	0.1	-
germacrene B	1561	1557	-	1.8	-	0.6
α-bisabolol	1685	1682	-	-	-	0.3
Total			97.2	95.7	99.4	94.8

KI cal.: Calculated Kovats indexes; KI lit.

### Anti-inflammatory activity

**Fig 1:**
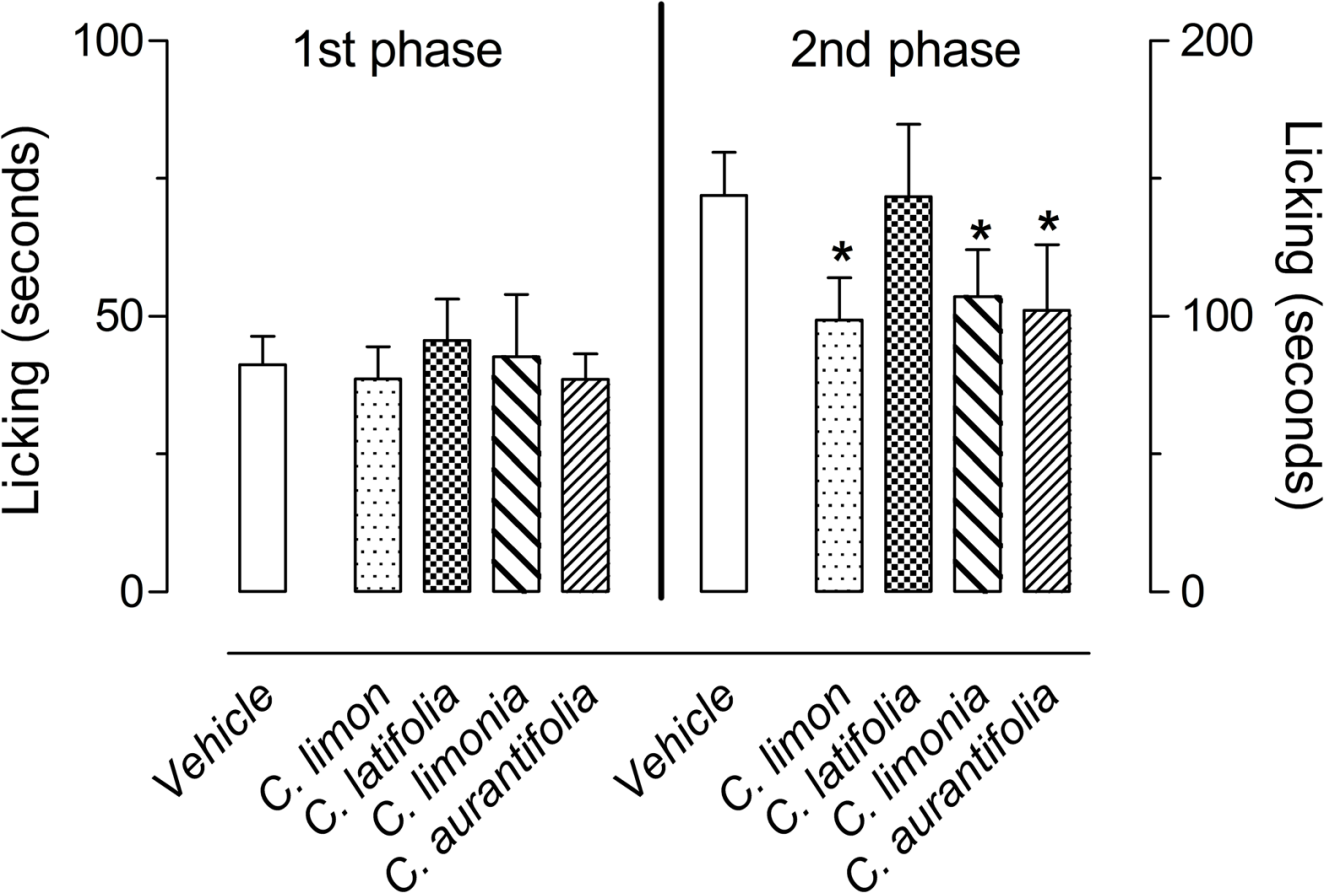
Effects of *C*. *limon*, *C*. *latifolia*, *C*. *limonia* and *C*. *aurantifolia* essential oils on the formalin-induced licking response in mice. Animals were pre-treated with oral doses of 100 mg/kg dose of each essential oil or vehicle. The results are presented as the mean ± S.D. (n = 6 per group) of the time that the animal spent licking the formalin-injected paw. Statistical significance was calculated by ANOVA followed by Bonferroni's test. *P < 0.05 when compared to vehicle-treated mice.

In order to evaluate possible anti-inflammatory or antinociceptive effects of the EOs obtained from *C*. *limon*, *C*. *aurantifolia*, *C*. *latifolia* and *C*. *limonia*, the first model used was the formalin-induced paw licking. After subplantar injection of formalin (2%), an intense licking behavior divided in two distinct phases was observed. The first phase developed during the first 5 minutes after formalin injection, with mice in the control group continuing to lick the paw for 41.3 ± 5 seconds. The second phase developed between 15 and 30 minutes after formalin injection, with mice continuing to lick the injected paw for 143.8 ± 15.7 seconds. None of EO inhibited the 1^st^ phase, while pre-treatment of the mice with 100 mg/kg of each EO resulted in a significant inhibition of the 2^nd^ phase of formalin-induced licking for the *C*. *limon*, *C*. *limonia* and *C*. *aurantifolia* EOs (Fig 1). To rule out a possible central antinociceptive activity from the EOs, we also evaluated their effects in the hot plate model. Even the highest dose (100 mg/kg) of each EO did not demonstrate any antinociceptive activity in this model, indicating that there is no central effect (S1 Fig).

**Fig 2:**
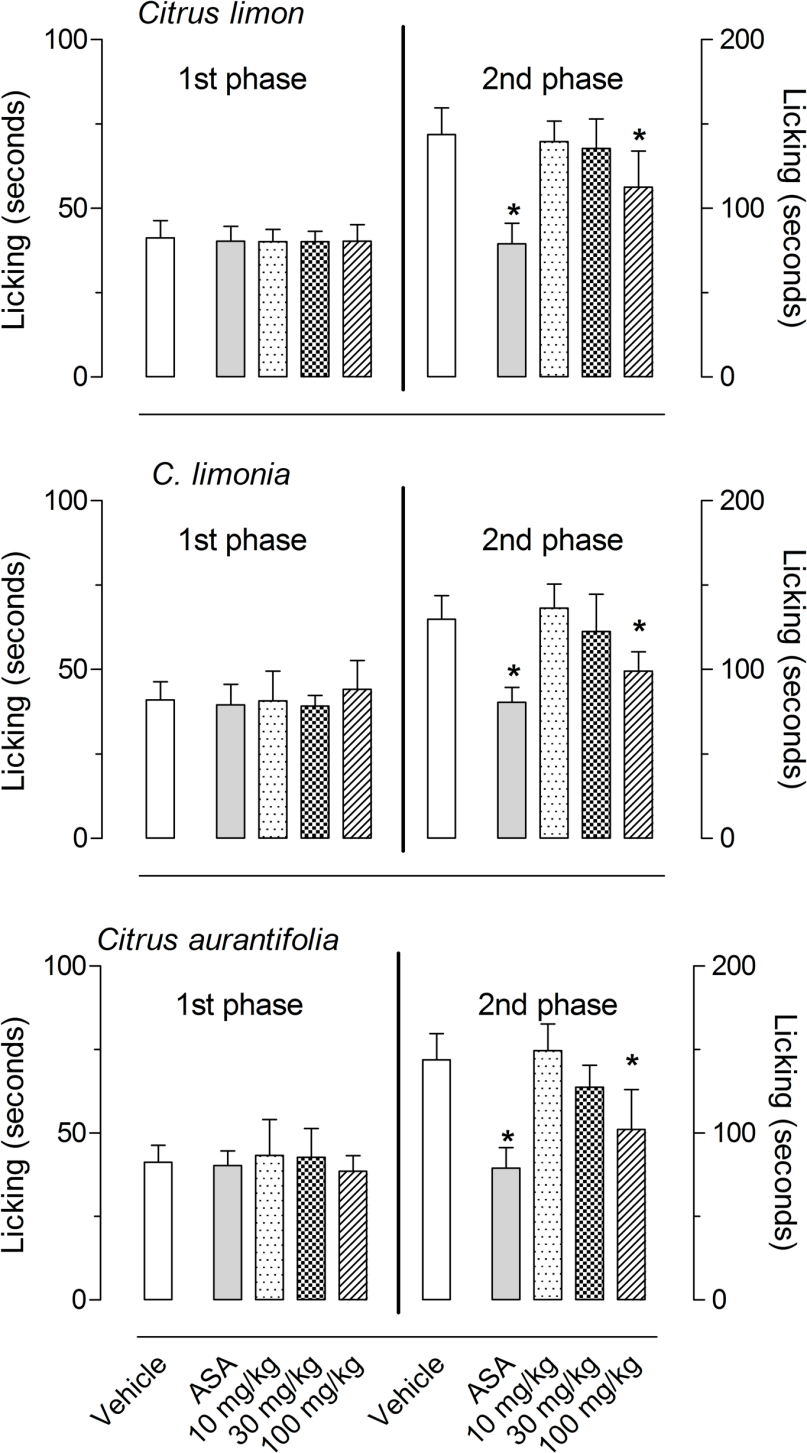
Effects of *C*. *limon*, *C*. *limonia* and *C*. *aurantifolia* essential oils on the formalin-induced licking response in mice. Animals were pre-treated with oral doses (10, 30 or 100 mg/kg) of each essential oil, acetylsalicylic acid (ASA, 100 mg/kg) or vehicle. The results are presented as the mean ± S.D. (n = 7 per group) of the time that the animal spent licking the formalin-injected paw. Statistical significance was calculated by ANOVA followed by Bonferroni's test. *P < 0.05 when compared to vehicle-treated mice.

Based on the results of the formalin-induced licking behavior, we decided to further test smaller doses of those EOs that demonstrated a significant effect (i.e., *C*. *limon*, *C*. *limonia* and *C*. *aurantifolia*). Fig 2 shows that all EOs reduced the licking response only at the higher dose (100 mg/kg). None of smaller doses (10 and 30 mg/kg) significantly inhibited the formalin response. The positive control group used (acetylsalicylic acid, ASA) significantly reduced licking-response at the 2^nd^ phase.

**Fig 3:**
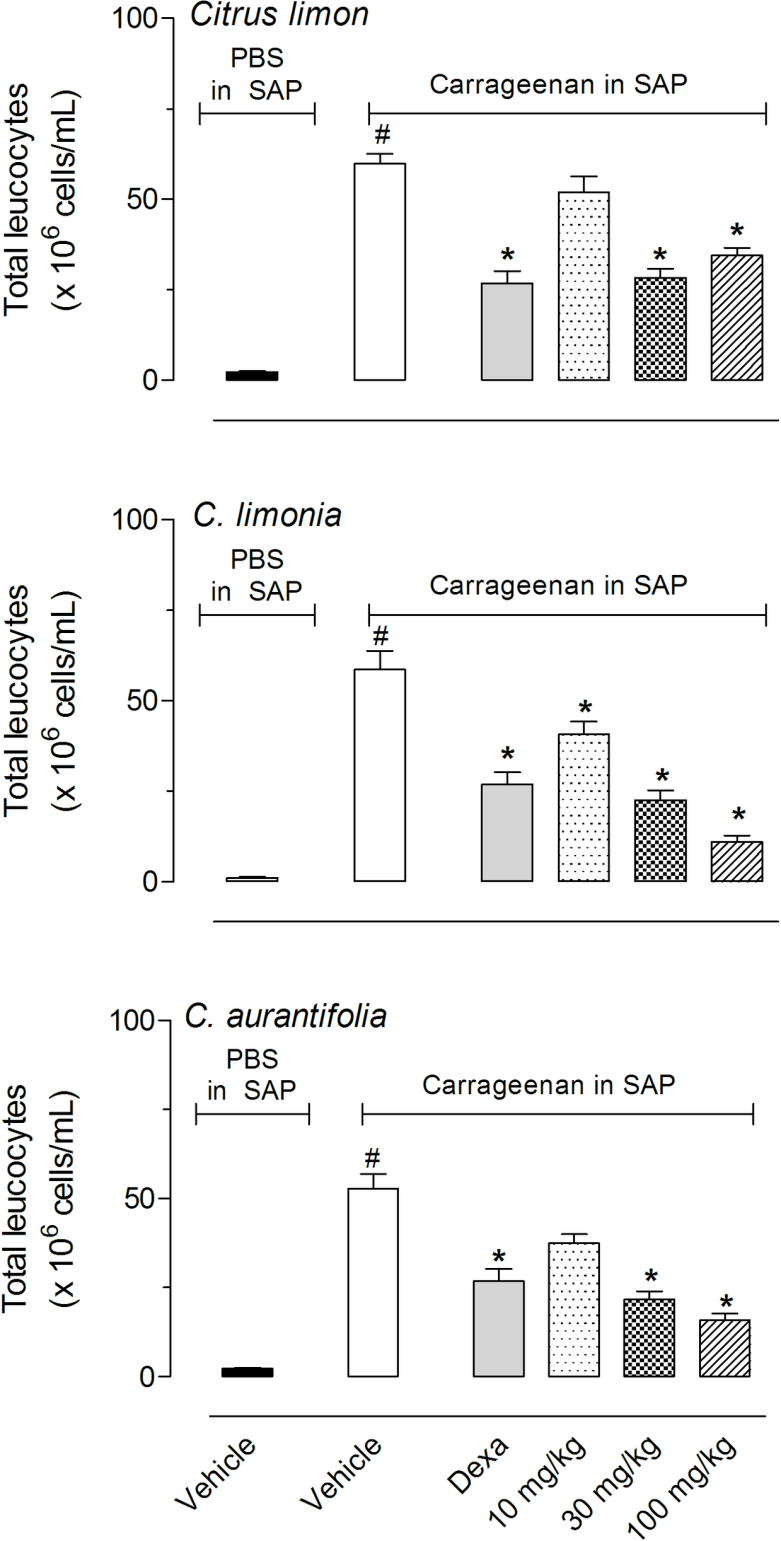
Effects of *C*. *limon*, *C*. *limonia* and *C*. *aurantifolia* essential oils on leukocyte migration into the subcutaneous air pouch (SAP). Animals were pretreated with different doses of the essential oils, dexamethasone (Dexa, 5 mg/kg, i.p.) or vehicle 1 h prior to carrageenan (1%) injection into the SAP. The results are presented as the mean ± S.D. (n = 10 per group) of cells (x 10^6^/mL) in the SAP. Statistical significance was calculated by ANOVA followed by Bonferroni's test. ^#^P < 0.05 when comparing vehicle treated group that received carrageenan in the SAP with vehicle-treated animals that received PBS in SAP; *P < 0.05 when comparing essential oils-treated animals with that received carrageenan in the SAP with the group that only received carrageenan in the SAP.

Next, we analyzed the capacity of the EOs to reduce cell migration into the subcutaneous air pouch (SAP) after the injection of carrageenan. The results obtained in this model show that *C*. *limon* and *C*. *aurantifolia* significantly reduced cell migration after pre-treatment of animals with 30 or 100 mg/kg, while *C*. *limonia* demonstrated a significant inhibitory effect with all three doses tested (10, 30 and 100 mg/kg). The effects observed with 30 or 100 mg/kg doses were similar to those obtained with the positive group, dexamethasone (Dexa, 1.5 mg/kg, i.p.) (Fig 3). The differential cell count of exudates demonstrated that more than 90% of leukocytes that migrate to the SAP was composed by polymorphonuclear neutrophils. Reduction in cells number inhibited both mononuclear and polymorphonuclear leukocytes without distinction between then (data not shown).

It seems that a maximal inhibitory effect occurs with 30 and 100 mg/kg doses since there were any statistical differences between these doses. For this reason we did not increase the doses more than 100 mg/kg.

**Fig 4:**
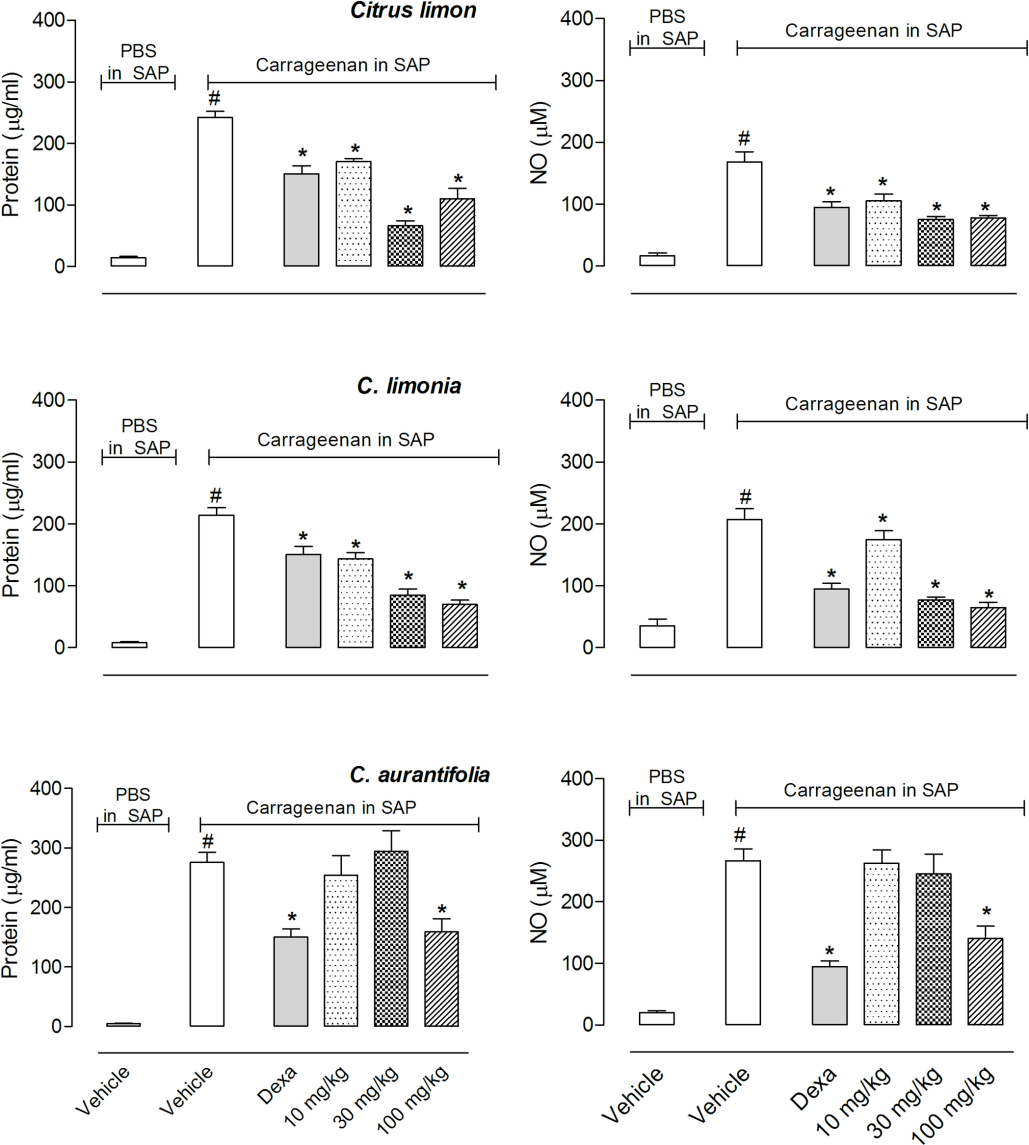
Effects of *C*. *limon*, *C*. *limonia *and *C*. *aurantifolia* essential oils on carrageenan-induced protein extravasation and nitric oxide (NO) production in a subcutaneous air pouch (SAP). Animals were pre-treated with various doses (10, 30 or 100 mg/kg, p.o.) of EO, dexamethasone (5 mg/kg, i.p.) or vehicle. The results are presented as the mean ± S.D. (n = 10 per group). Statistical significance was calculated by ANOVA followed by Bonferroni’s test. ^#^*P* < 0.05 when comparing the carrageenan-injected group with the PBS-injected group and *P < 0.05 when comparing EO or dexamethasone-treated groups with the vehicle-treated group.

Because both EOs significantly reduced cell migration into the SAP, we decided to further analyze other parameters present in the inflammatory processes induced by carrageenan. We therefore measured the amount of nitric oxide (NO) produced and the amount of protein extravasated to the exudate in the cavity. Both *C*. *limon*, *C*. *limonia* and *C*. *aurantifolia* EOs significantly reduced the amount of protein extravasated and NO produced in all three doses evaluated with results similar to those obtained after pretreatment of animals with dexamethasone (Fig 4).

**Fig 5:**
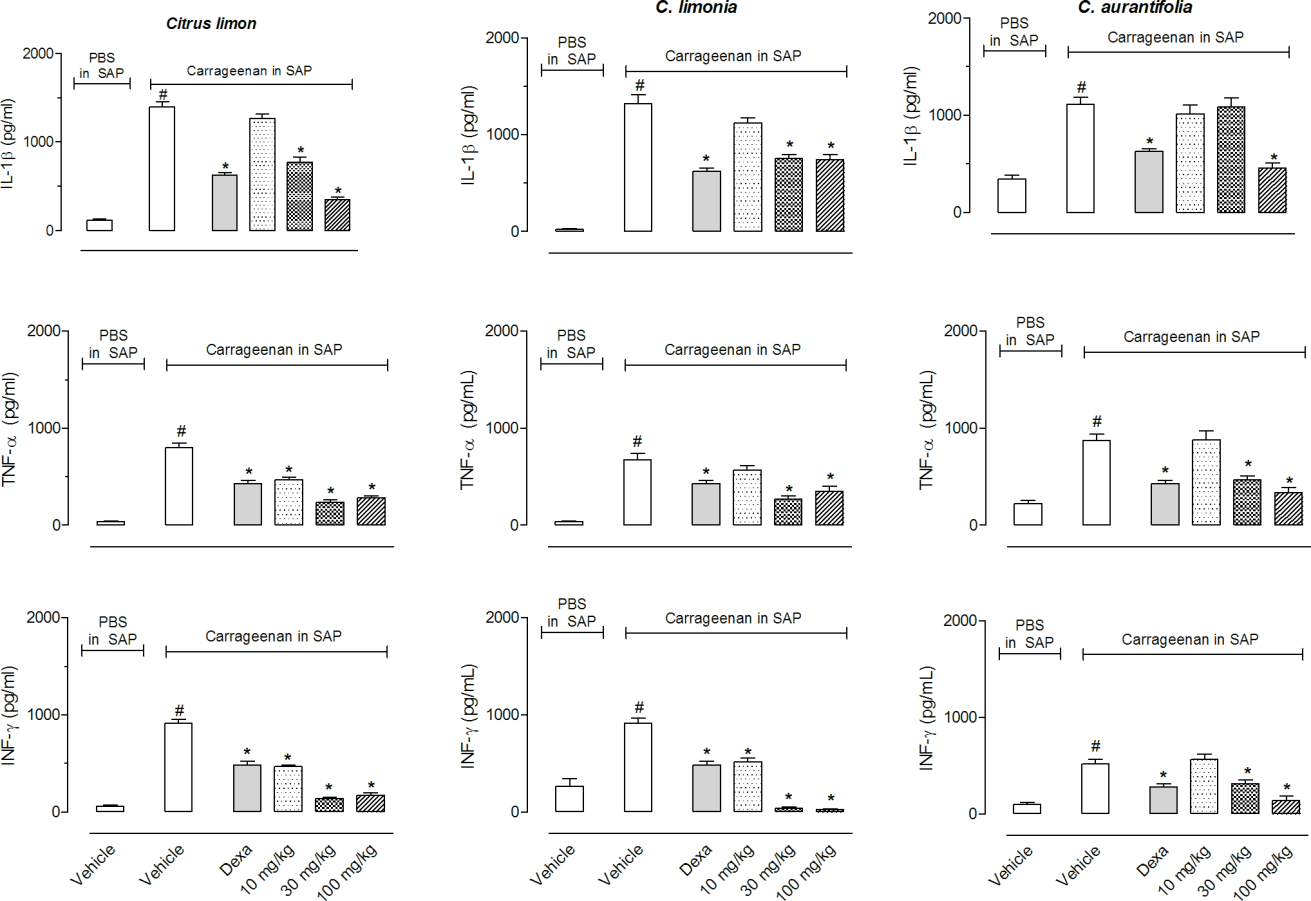
Effects of *C*. *limon*, *C*. *aurantifolia* and *C*. *limonia* essential oils on carrageenan-induced IL-1β, TNF-α and IFN-γ production in a subcutaneous air pouch (SAP). Animals were pre-treated with various doses (10, 30 or 100 mg/kg, p.o.) of EO, dexamethasone (5 mg/kg, i.p.) or vehicle. The results are presented as the mean ± S.D. (n = 10 per group). Statistical significance was calculated by ANOVA followed by Bonferroni’s test. ^#^*P* < 0.05 when comparing the carrageenan-injected group with the PBS-injected group and *P < 0.05 when comparing EO or dexamethasone-treated groups with the vehicle-treated group.

Fig 5 shows the results of the cytokine quantification in the SAP exudate. Carrageenan injection in the SAP induced 11.6, 22 and 15.4-fold increases in cytokines levels. Pretreatment with 30 or 100 mg/kg dose of *C*. *limon* significantly reduced the amount of IL-1β in the SAP, and all three doses inhibited TNF-α and IFN-γ production. *C*. *limonia* pretreatment reduced IL-1β and TNF-α production at 30 or 100 mg/kg doses, while IFN-γ production was reduced with all three doses and almost totally blocked with the highest doses (30 and 100 mg/kg). Levels of IL-1β in the exsudates were reduced with pretreatment of mice with higher dose of *C*. *aurantifolia* EO (100 mg/kg). While, doses of 30 and 100 mg/kg of this EO significantly reduced levels of TNF-α and IFN-γ. Pretreatment of mice with dexamethasone also significantly reduce cytokine levels.

**Fig 6:**
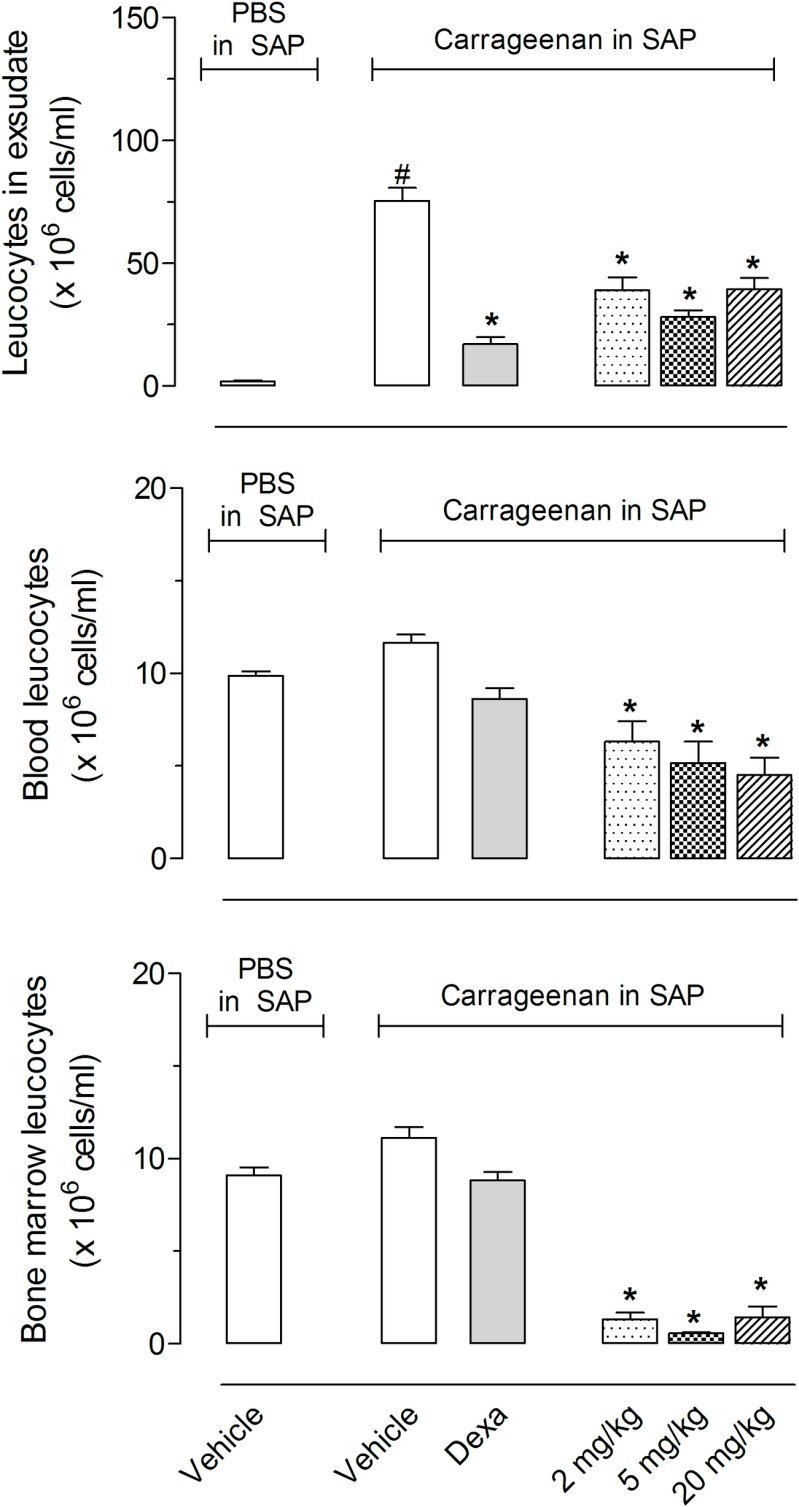
Effects of pure citral on total leukocyte counts in blood and bone marrow. Animals were pretreated with citral (2, 5 or 20 mg/kg) or vehicle 1 h prior to carrageenan (1%) injection into the SAP. The results are presented as the mean ± S.D. (n = 10 per group) of leukocytes (x 10^6^/mL) in ex udate, blood or bone marrow. Statistical significance was calculated by ANOVA followed by Bonferroni's test. *P < 0.05 when comparing citral-treated animals with carrageenan injected in the SAP with the group that received carrageenan in the SAP.

As the EOs significantly reduced cell migration into the SAP, we decided to evaluate a possible cytotoxic effect against blood and bone marrow leukocytes. There was no alteration in the number of leukocytes counted, even 24 h after oral administration of dexamethasone or 100 mg/kg *C*. *limon* or *C*. *limonia*. However, a significant reduction in leukocytes counts in blood and bone marrow were observed after treatment with the same dose of *C*. *aurantifolia* EO, suggesting cell toxicity (Fig 6).

The unexpected effect observed in *C*. *aurantifolia* led us to investigate the possible causes. As previously shown, all three EOs contain citral. *C*. *aurantifolia* EO contain 9.6% of the trans isomer (or geranial) and 7.1% of the cis isomer (or neral), for a total of 16.7% citral. *C*. *limon* EO contains 2.2% geranial and 1.7% neral for a total of 3.9% citral. To confirm that citral is responsible for the observed cytotoxicity, we tested purified citral obtained from the EO in doses of 2, 5 and 20 mg/kg. These doses corresponded to the amount obtained from *C*. *aurantifolia* EO. As can be observed in Fig 6, all three doses of citral significantly reduced the number of leukocytes in blood and bone marrow.

Due to the cytotoxic effect against the blood and bone marrow cells we decided to evaluate the possible toxicity in behavioral parameters in mice. After treatment with 500 mg/kg of EO we did not observe neither behavioral alterations nor stomachs lesions in the animals up to the oral dose of 500 mg/kg of body weight (data not shown).

**Fig 7:**
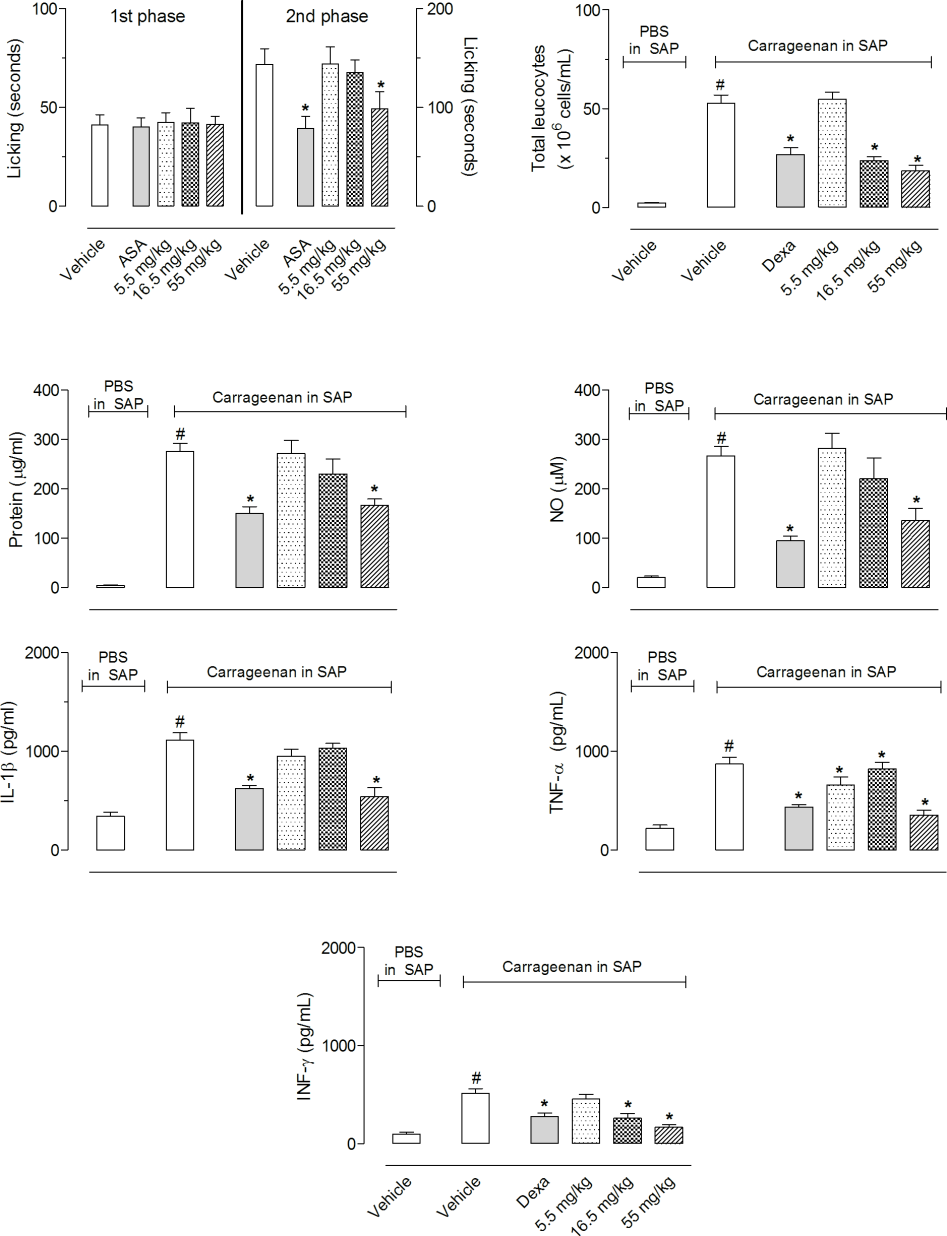
Effect of limonene on formalin-induced licking and SAP models. Animals were pretreated with various doses of limonene (5.5, 16.5 or 55 mg/kg) or vehicle 1 h prior to formalin (1%) or carrageenan (1%) injection. The results are presented as the mean ± S.D. (n = 6 per group). Statistical significance was calculated by ANOVA followed by Bonferroni's test. *P < 0.05 when comparing essential oils-treated animals with carrageenan injected in the SAP with the group that received carrageenan in the SAP.

After finding high amounts of limonene in the EOs (53.9% in *C*. *limon*, 65.7% in *C*. *limonia* and 31.1% in *C*. *aurantifolia*), we decided to evaluate pure limonene. The doses used were similar to the amount found in *C*. *aurantifolia* EO. Fig 7 show that pure limonene significantly reduced the formalin-induced licking behavior only in high doses (55 mg/kg). In the SAP model, 16.5 and 55 mg/kg doses reduced cell migration to and IFN-γ production in the pouch, whereas protein extravasation, NO and IL-1β production were reduced only with a 55 mg/kg dose. TNF-α levels were inhibited by all three doses (5.5, 16.5 and 55 mg/kg). None of the doses tested influenced the total leukocyte counts in blood or bone marrow (data not shown).

## Discussion

In this study we demonstrated that essential oils from several citrus species presented significant anti-inflammatory effects in different models in mice. To confirm the hypothesis that essential oil (EO) from *C*. *limon*, *C*. *latifolia*, *C*. *aurantifolia* and *C*. *limonia* have antinociceptive effects, we tested each one on the formalin-induced licking behavior. This model is a widely-used pain model in evaluating antinociceptive and anti-inflammatory drugs. There are two phases, with the first (neurogenic phase) occurring peripherally and resulting from formalin activation of nociceptors located in the tissue and the second (inflammatory phase) occurring after the release of a multitude of molecules, such as histamine, serotonin, and bradykinin, developing an inflammatory response [25]. Our results suggest that Eos from *C*. *limon*, *C*. *aurantifolia* and *C*. *limonia* have an anti-inflammatory effect because they reduced the second phase response to formalin. This may occur through a reduction in inflammatory mediator liberation in mice paws or a direct action on one or more mediator receptors.

The formalin model alone cannot be used to ascertain the anti-inflammatory effects of the *Citrus* species. We therefore used the carrageenan-induced inflammation in a subcutaneous air pouch (SAP) model. This model is characterized by a drastic increase in leukocytes, cytokines, inflammatory mediators and protein after the carrageenan injection [20]. The mechanism by which carrageenan induces the inflammatory response is complex and involves the liberation of several mediators and an increase in vascular permeability. Our results indicate that EOs induce an anti-inflammatory effect by reducing several of the parameters observed in carrageenan-induced inflammation. These results cannot guarantee the exact local action of the EOs, but do suggest that EOs can be acting in one or more mediator systems involved in the inflammation.

During the inflammatory event there is also an increase in cytokine production. Tumor necrosis factor-α (TNF-α), interleukin-1β (IL-1β), and interferon-γ (IFN-γ) have important roles in the maintenance of the inflammatory profile [26, 27]. Our results indicate that EOs have significant anti-inflammatory effects in reducing all parameters evaluated. Since IL-1β and TNF-α regulate leukocyte migration, we can infer that a reduction in leukocyte number may have been a direct effect of EOs as well as an indirect effect resulting from the reduction in the levels of those cytokines. Taken together, these results indicate that these EOs act similarly to immunomodulators in reducing cell migration and inflammatory mediator production. Our results are in agreement with others that showed various essential oils or their constituents inhibit cytokine production. For example, 1,8-cineol inhibited TNF-α and IL-1β in human lymphocytes, α-humulene reduced TNF-α production, and terpinen-4-ol suppressed the production of TNF-α, IL-1β, IL-8, IL-10, and PGE2 by LPS-activated monocytes [28]. *C*. *latifolia* reduced TNF-α and IL-10 levels in zymosan-induced peritonitis [9].

Despite all EOs present limonene in there constitution a difference occurred between the inhibitions observed in the formalin-induced licking and carrageenan-induced cell migration models. A possible explanation to the differences could be the fact that both models are very different. In the first one we observe liberation of inflammatory mediators such as histamine, serotonin and bradykinin in mice paws [17]. In the second model we have the involvement of several other mediators and systems, liberation of nitrogen reactive species (ROS) and cytokines [19]. In this regard, one possible explanation to the fact that EOs did not demonstrate a significant inhibitory effect in the 2^nd^ phase of formalin-induced licking model despite its content of limonene could be explained by the possibility that limonene has a more pronounced effect in cells activities, cytokine and/or ROS production and not in mediators that are mainly released in the formalin-induced licking response.

Additionally, *C*. *aurantifolia* EO demonstrated some toxicity, likely due to the presence of high amounts of citral. Others have demonstrated a toxic effect of citral. However, those groups used different models such as embryofetal [29] and carcinogenesis induction [30]. Although very different models and doses were used, our data, in our experimental conditions, indicate that citral has toxic effect in other model and indicate that this effect is not a model-specific effect. With our acute assays it is not possible to definitively conclude that there is a myelotoxicity given that such effects are usually studied in repeated dose toxicity studies, as e.g. given in the FDA guideline. It seems premature to conclude definitively that there is myelotoxicity also in other conditions. The observed phenomena could also be a temporary reaction which would resume later, so that further studies in models for repeated dose toxicity and e.g. also in another animal species seem adequate. In this regard, further studies are necessary before drawing so far reaching conclusions, given the wide spread occurrence of citral in many plants as in food as in medicinal use.

We further showed that *C*. *limon* and *C*. *limonia* have significant effects, likely due to the presence of high amounts of limonene (53.9% and 31.1%, respectively). The fact that EO from *C*. *limon* and *C*. *limonia* presented significant anti-inflammatory effect probably due to the presence of limonene (53.9% and 31.1%, respectively) and EO from *C*. *latifolia* did not present this effect even with 35.4% limonene could be explained, at least in part, by an assumption that some chemical interactions may be occurring in intestinal tract in such a way that this EO did not present a good absorption. Or it could be that any other substance present in a different amount than in others EO could interfere with an effect. Several pharmacological effects for limonene have been documented, such as *in vitro* inhibition of NO and PGE2 production [31], antineoplastic [32] and anti-inflammatory in a colitis model [33], but to the best of our knowledge this work is the first to describe its effect as an inhibitor of cell migration and cytokine production.

## Conclusions

Our results indicate that the essential oils obtained from *Citrus limon*, *Citrus limonia* and *Citrus aurantifolia* demonstrate a significant anti-inflammatory effect. We also draw attention to the fact that *C*. *aurantifolia* is rich in citral, which gives it a toxic and myelotoxic effects. In this regard, care should be taken with *C*. *aurantifolia* due to its potential toxic effect. The essential oils compositions found for the species included in this report are inside the range of compositions already published for those species [3]. Additionally all the species are cultivated, meaning that we have homogenous genetic matrices as oil sources. However, due to the well-recognized variability in the composition of secondary metabolites caused by the susceptibility of plants to, for example, seasonal, geographical and geographical influence the potential applicability of these oils to treat inflammatory conditions would gain efficacy and safety after the standardization of production of these oils focusing on the best composition for therapeutic applications.

## Supporting Information

S1 Fig Effects of *C*. *limon*, *C*. *latifolia*, *C*. *limonia* and *C*. *aurantifolia* essential oils on the hot plate model. Animals were orally pretreated with different doses of each essential oil or vehicle. The results are presented as mean ± S.D. (n = 6 per group) of the increase in response time relative to baseline levels. Statistical significance was calculated by ANOVA followed by Bonferroni's test.
(TIF)
