# Supplementary figures and images for: Anti-Inflammatory Properties and Chemical Characterization of the Essential Oils of Four Citrus Species
Source: PLoS One. 2016 Apr 18;11(4):e0153643. doi: 10.1371/journal.pone.0153643 (PMC4835072; doi:10.1371/journal.pone.0153643)

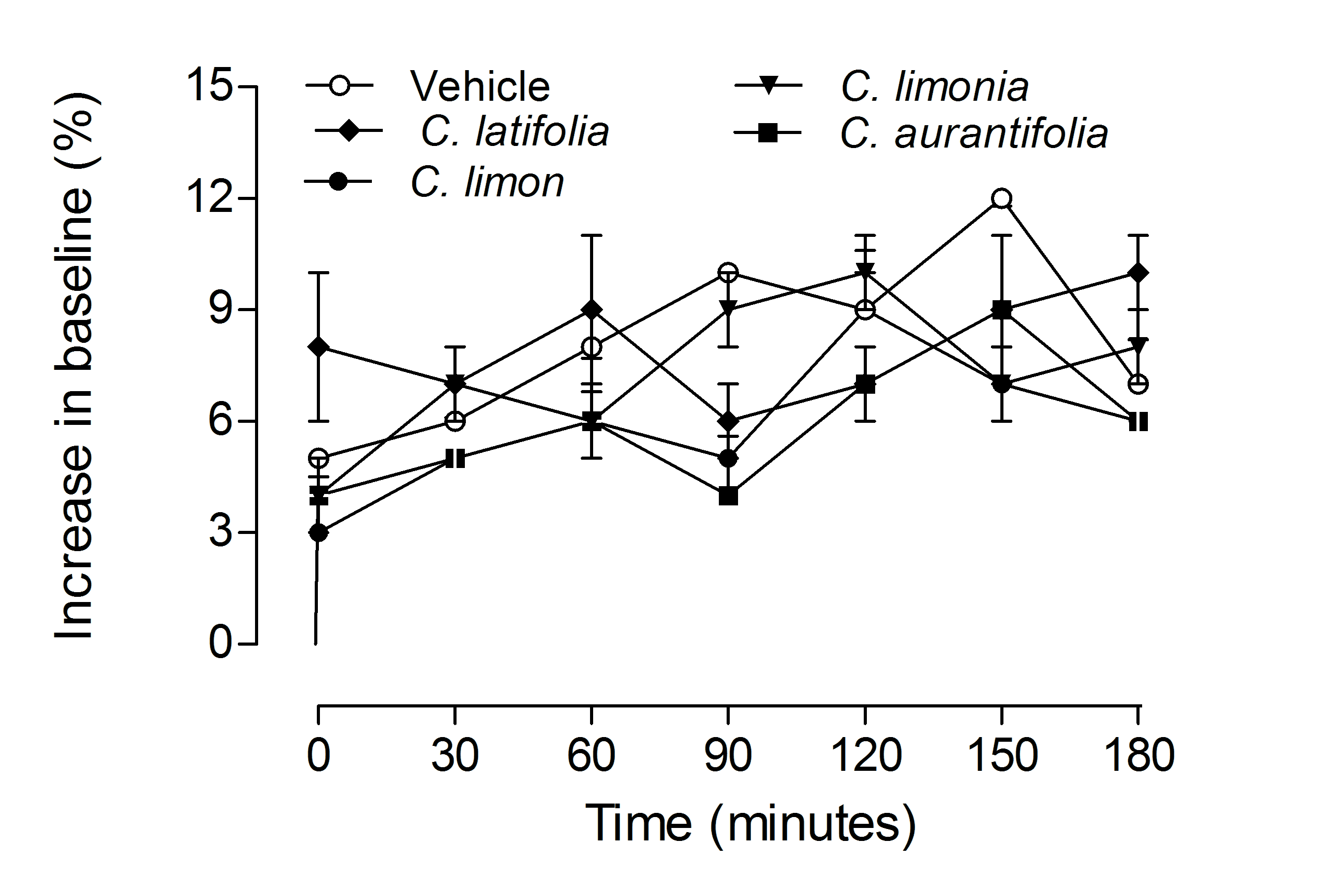

Supplement: S1 Fig — Animals were orally pretreated with different doses of each essential oil or vehicle. The results are presented as mean ± S.D. (n = 6 per group) of the increase in response time relative to baseline levels. Statistical significance was calculated by ANOVA followed by Bonferroni's test. (TIF) [file pone.0153643.s001.tif]
